# Thermal deformation of cryogenically cooled silicon crystals under intense X-ray beams: measurement and finite-element predictions of the surface shape

**DOI:** 10.1107/S0909049513009436

**Published:** 2013-05-08

**Authors:** Lin Zhang, Manuel Sánchez del Río, Giulio Monaco, Carsten Detlefs, Thomas Roth, Aleksandr I. Chumakov, Pieter Glatzel

**Affiliations:** a European Synchrotron Radiation Facility, 6 rue Jules Horowitz, BP 220, Grenoble 38043, France

**Keywords:** silicon crystal, X-ray monochromator, cryogenic cooling, liquid nitrogen, thermal deformation, finite element, heat load experiment, rocking curve, *in situ* metrology

## Abstract

X-ray crystal monochromators exposed to white-beam X-rays in third-generation synchrotron light sources are subject to thermal deformations that must be minimized using an adequate cooling system. A new approach was used to measure the crystal shape profile and slope of several cryogenically cooled (liquid nitrogen) silicon monochromators as a function of beam power *in situ* and under heat load. The method utilizes multiple angular scans across the Bragg peak (rocking curve) at various vertical positions of a narrow-gap slit downstream from the monochromator. When increasing the beam power, the surface of the liquid-nitrogen-cooled silicon crystal deforms from a concave shape at low heat load to a convex shape at high heat load, passing through an approximately flat shape at intermediate heat load. Finite-element analysis is used to calculate the crystal thermal deformations. The simulated crystal profiles and slopes are in excellent agreement with experiments. The parameters used in simulations, such as material properties, absorbed power distribution on the crystal and cooling boundary conditions, are described in detail as they are fundamental for obtaining accurate results.

## Abstract 2

The shape of cryogenically cooled monochromator crystals deformed by the heat load of the X-ray beam is derived from rocking curve measurements at various vertical positions of a narrow-gap slit downstream from the monochromator. Experimentally, it is observed that the crystal shape changes from concave to convex when beam power increases. The observations are accurately modelled by finite-element analysis, showing an excellent quantitative agreement with experiments.

## 1. Introduction   

Perfect silicon single crystals are widely used as X-ray monochromators at third-generation synchrotron light sources. The beam footprint on the monochromator crystal (X-ray beam illuminated area) is variable and typically much smaller than the crystal size because of the variable and large Bragg angle. The beam power density on the crystal can reach several hundred W mm^−2^. Under these circumstances, liquid-nitrogen (LN2) cooling is the most effective way to limit thermal deformations. LN2-cooled silicon crystals have been widely used with great success at many synchrotron light sources (Marot *et al.*, 1992 ▶; Rogers *et al.*, 1995 ▶; Lee *et al.*, 1995 ▶; Marot, 1995 ▶; also see the reviews by Bilderback *et al.*, 2000 ▶; Mochizuki *et al.*, 2001 ▶; Zhang *et al.*, 2003 ▶; Chumakov *et al.*, 2004 ▶). Numerous studies have been carried out to assess the performance limits of cryogenically cooled silicon monochromators both by finite-element analysis (FEA) modelling (Zhang, 1993 ▶; Zhang *et al.*, 2003 ▶) and experimental testing (Lee *et al.*, 2000 ▶, 2001 ▶; Tajiri *et al.*, 2001 ▶; Zhang *et al.*, 2003 ▶; Chumakov *et al.*, 2004 ▶). FEA simulations can determine the strain field in a heat-distorted crystal, and therefore the deformed shape of the crystal. The thermal deformation of the crystal induces rocking curve broadening, which leads to a loss of monochromatic flux. Furthermore, the shape change of the reflecting surface may deform the wavefront and thus have a negative impact on coherent and micro-focusing experiments. Additional deformations may arise, *e.g.* from strains introduced by the mechanical mounting of the crystal. The effective width of the rocking curve can be estimated, to a first approximation, by adding in quadrature the peak-to-peak slope error of the deformed crystal (including thermal deformation calculated by FEA and initial mechanical mounting deformation) with the theoretical intrinsic diffraction width (Zhang *et al.*, 2003 ▶). Most studies in the literature compare the rocking curve width calculated in this way with the measured one. However, these papers present an indirect comparison between FEA results in thermal deformation and measurement results in the rocking curve broadening. The measured rocking curve broadening is balanced between two important facts. On one hand there is a change of shape of the crystal surface owing to the thermal deformations. This important geometrical effect (modification of the reflecting surface) is always present and it is discussed in detail in this paper. On the other hand, the thermal load produces a distortion of the diffracting crystal volume (thermal stress) that may, in cases of high thermal load, significantly modify the diffracting properties of the crystal, ranging from dynamic diffraction (no distortion of small distortions) to kinematical diffraction. For small thermal stress of the crystal (LN2-cooled silicon crystal with moderate heat load or water-cooled crystal with very low heat load), the geometrical approach is sufficient (Zhang *et al.*, 2001 ▶, 2003 ▶; Mocella *et al.*, 2001 ▶; Hoszowska *et al.*, 2001 ▶). However, high heat loads produce a significant distortion of the crystal lattice, and the diffraction profile differs substantially from that of the undistorted crystal. In order to match FEA predictions with experimental rocking curves, it is necessary to solve the Takagi–Taupin equations (Takagi, 1962 ▶, 1969 ▶; Taupin, 1964 ▶, 1967 ▶) using the strain distribution in the crystal calculated by FEA. This approach has been successfully applied to water- or liquid-nitrogen-cooled monochromators (Zhang *et al.*, 2001 ▶; Mocella *et al.*, 2001 ▶, 2003 ▶; Hoszowska *et al.*, 2001 ▶) where a good agreement was reported between calculated and measured rocking curves.

In order to preserve the characteristics of the photon beam in terms of flux and divergence, the design of the monochromator and cooling system has to be optimized so that the thermal slope error of the monochromator is much smaller than the angular width of the reflectivity curve (Darwin width). The ongoing European Synchrotron Radiation Facility (ESRF) Upgrade Programme (2009–2015) includes the design and construction of new beamlines [UPBLs (upgrade beamlines)], and the refurbishment and upgrade of existing beamlines. The beamline performances will be significantly improved and nano-focused beams will be routinely used owing to advances in undulator sources and beamline optics technologies. More optical elements in addition to the monochromator will be used in beamlines both upstream (white-beam mirrors) and downstream (Kirkpatrick–Baez mirrors, transfocators combining several compound refractive lenses *etc.*) from monochromators. Changes in the beam divergence induced by thermally deformed monochromator crystals must be considered globally as they affect the performance of the other optical elements. The overall beamline performance depends not only on the thermal slope error related to the rocking curve broadening for the photon flux preservation, but also on the thermally deformed crystal shape for the beam collimation, focusing and preservation of coherence. Therefore, in the design and optimization of the beamline optics it is essential to have accurate and reliable predictions of the shape of the optical elements under heat load. The information gained until now from the comparison between rocking curve measurements and thermal deformation calculations by FEA is insufficient to define to the specifications required for high-performance beamline design and optimization. The method presented here allows the direct experimental determination of the crystal’s local thermal deformation and thus provides a more stringent test for FEA simulations.

As an example, the ESRF UPBL6 [Inelastic X-ray Scattering (IXS)] beamline requires an excellent collimation upstream of a high-resolution monochromator. The heat load on the pre-monochromator has to be considered together with the performance of a white-beam collimating mirror, as these two optical elements absorb almost all of the beam power. To keep the collimation to low values, the shape of both the mirror and pre-monochromator under heat load need to be simulated reliably and accurately. In general, the performances of the optics downstream from a crystal monochromator depend on the characteristics of the beam provided by the monochromator, that are in turn defined by its thermally deformed shape.

In order to provide a direct comparison between the FEA and experiments on the thermal deformation of the Si crystals, we have performed simultaneous heat load experiments on LN2-cooled Si crystal monochromators on several ESRF beamlines under various heat load conditions. This paper presents the experimental set-up and measurement results on the thermal slope error profile, reviews the FEA simulation of the LN2-cooled Si crystal, discusses the particularities of the FEA inputs used at the ESRF, and compares the FEA simulations with the experimental results.

## 2. Experimental   

### 2.1. Beamline set-up   

Heat load experiments on LN2-cooled Si monochromator crystals at the ESRF have been conducted simultaneously at three ESRF beamlines: ID06 (Techniques and Instrumentation Test beamline), ID18 (Nuclear Resonance beamline) and ID26 (X-ray Absorption and Emission Spectroscopy beamline). To vary the beam power without changing the other characteristics of the X-ray beam, the electron beam current in the storage ring was ramped from 0 to 300 mA in steps of 50 mA, passing through 200 mA (presently the most common operation current). Two dedicated experimental sessions were allocated to these heat load tests. Two beamlines (ID06, ID18) are equipped with a 0.3 mm diamond window while ID26 is windowless. All beamlines have water-cooled primary slits and a LN2-cooled double silicon crystal monochromator. At the ID26 beamline there is also a water-cooled white-beam mirror upstream of the double-crystal monochromator (DCM). The white-beam mirror reduces the heat load on the DCM, but could affect the beam divergence. Therefore, it is more appropriate to concentrate on the DCMs exposed to white beam in order to assess the performance of the LN2-cooled silicon crystal monochromator. For the quantitative results presented here we use data from beamlines ID06 and ID18, though the measurements carried out at ID26 are in good qualitative agreement with the other results.

**Figure 1:**
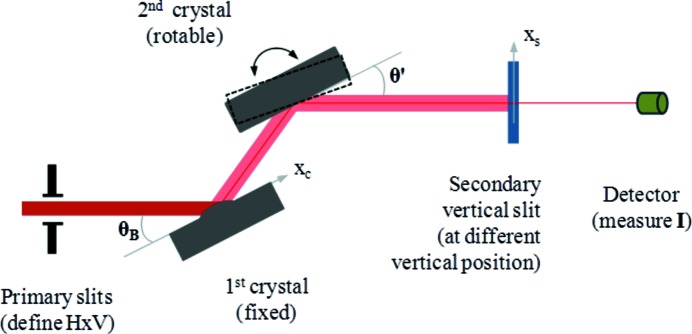
Set-up scheme for the thermal deformation profile measurements using rocking angle scans at various vertical positions of a narrow-gap exit slit. The rocking angle θ′ corresponding to the peak intensity in the rocking curve for the distorted first crystal is slightly different from the Bragg angle θ_B_ (peak position for the undistorted crystal). The distortion of the crystal in terms of angle is given by Δθ = θ′ − θ_B_. Each slit position *x*_s_ (*x*_s_ = 0 for the centre of the beam) allows the rocking curve to be recorded relative to the X-ray beam impinging on the first crystal surface at the position *x*_c_ = *x*_s_/sinθ_B_. The thermal slope distribution Δθ(*x*_c_) of the crystal can be measured by varying the vertical positions of a narrow-gap slit after the monochromator.

The schematic experimental set-up at beamlines ID06 and ID18 is shown in Fig. 1 ▶. There are two undulators at beamline ID06: an in-vacuum undulator U18 and a conventional undulator U32. The in-vacuum undulator U18 was set to a 8.3 mm gap in order to have the fundamental energy at *e*_1_ = 13.848 keV, and the undulator U32 to a 13.55 mm gap in order to have the third harmonics at the same photon energy. In addition to the 0.3 mm-thick diamond window separating the storage ring and the beamline, there are two beryllium windows with a total thickness of 1 mm at beamline ID06 upstream of the monochromator. The experimental set-up at beamline ID18 is quite similar. Three U20 undulators are used with gaps set to obtain the fundamental photon energy at *e*_1_ = 14.413 keV. The characteristics of the undulators, the beamline settings and the calculated power for a primary slits opening of 2 mm × 1 mm (H × V) at 200 mA electron beam current are summarized in Table 1 ▶. The primary slits, upstream of the monochromator, define the beam size on the monochromator and have a fixed opening [mostly 2 mm × 1 mm (H × V)] to maintain a constant heat load profile during the experiments. The secondary slits, after the monochromator, are set to a horizontal narrow gap (50 µm), and moved vertically to isolate rocking curve data to a specific striped area on the surface of the first crystal.

The DCMs used for these tests allow the second crystal to be scanned while keeping the first one fixed. As the first crystal absorbs almost all of the incident power, the heat load on the second crystal is insignificant for the thermal deformation. Therefore the second crystal is supposed to be non-deformed with an ideal flat shape. The increase of the rocking curve width (measured after the secondary slits) for a distorted first crystal with respect to the undistorted flat crystal provides an estimate of the global slope error (peak-to-peak slope error). In order to measure the local slope error distribution on the first crystal surface, a series of rocking curves were measured at different vertical positions of the narrow-gap slit after the monochromator. Each slit position *x*_s_ (*x*_s_ = 0 for the centre of the beam) allows the recording of the rocking curve related to the photons impinging on the first crystal surface at the position *x*_c_ = *x*_s_/sinθ′, where θ′ = θ_B_ + Δθ, θ_B_ is the Bragg angle and Δθ the distortion of the crystal in terms of angles (see Fig. 1 ▶). These angles can be determined from the rocking curve measurements: θ′, the rocking angle at which the rocking curve reaches maximum for a distorted crystal; θ_B_, the peak position for the undistorted flat crystal. For a LN2-cooled silicon crystal, the thermal slope error Δθ is of the order of 10 µrad which is much smaller than the Bragg angle θ_B_ (25.4° for the ID06 monochromator). The exit beam after the monochromator is parallel to the incident beam owing to the double crystals. The thermal deformation of the first crystal can induce a parallel beam displacement δ*x*_s_ at the position of the scanning slit. This displacement can be calculated as δ*x*_s_ = *L*Δθtan(θ_B_), where *L* is the distance between the exit beam and incident beam, and approximately equal to 15 mm. For Δθ = 10 µrad of thermal slope error, the beam position shift δ*x*_s_ ≃ 0.071 µm is much smaller than the scanning slit gap (50 µm), and independent of the distance between the scanning slits and the monochromator. An advantage of this method is to only use the peak positions of the different rocking curves. Therefore it is independent of the diffraction profile so it also works for the case of large thermal deformation where diffraction profiles are usually calculated by Takagi–Taupin equations. By varying the vertical position of the narrow-gap slit, we obtain the angular distribution Δθ(*x*_c_), also called the thermal slope error distribution. The integral of this angular distribution gives the deformation profile; the derivative of this angular distribution gives the curvature (approximately the inverse of the radius) distribution. In summary, the traditional rocking curve width measurement with full beam gives only the rocking curve broadening, providing indirect and global information on the thermal deformation, while measurement of the rocking curve peak angular position through a vertical narrow-gap slit at different slit positions enables the slope profile of the deformed crystal to be recorded, thereby providing direct and local information on the thermal deformation.

### 2.2. Calorimetry description   

The thermal and mechanical properties of silicon *versus* temperature are strongly non-linear in the LN2 temperature range. The temperature and thermal deformation of the LN2-cooled Si crystal are very sensitive to the heat load. Therefore, for FEA simulations it is essential to use very accurate values of the power from the beam used in the experiment. To fulfil this requirement, a calorimeter was developed at the ESRF to measure the X-ray beam power (Zhang & Biasci, 2005 ▶). It consists of a 100 mm × 50 mm × 50 mm copper block with a hole of 10 mm in diameter and 70 mm deep, through which the X-ray beam is sent. In this geometry the power losses by scattering outside the copper block are negligible. The calorimeter is mounted in a vacuum vessel, supported by a thermal insulator and was not cooled during the measurement. A Pt-100 temperature sensor attached to the cooper block is used to measure the temperature response of the calorimeter. Initially, the temperature *T*_0_ of the copper block is uniform. The X-ray beam was sent into the hole for approximately Δ*t* = 60 s and then switched off by a fast shutter. The temperature measured at the position of the Pt-100 sensor stabilizes at a constant value *T*_m_ about 120 s after the beam has been switched off. During this short period the energy loss is less than 0.6% of the total incident beam energy. Therefore, the uniform temperature *T*_m_ is practically the average temperature of the calorimeter at the moment the beam is switched off. The average beam power *P* can be calculated from the temperature increase during this period Δ*T* = *T*_m_ − *T*_0_ as
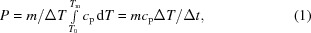
where *m* is the mass of the copper block and *c*_p_ is the heat capacity of copper. A single power measurement takes about 180 s using this method. Taking into account the temperature measurement accuracy and the power loss, the accuracy of the calorimeter is estimated to be in the range 0 to −1.2%.

### 2.3. Results   

#### 2.3.1. Beam power and calorimetry   

In order to calculate the thermal deformations of the monochromator crystals, the power distribution of the incident white beam must be known. The photon flux and power produced by storage rings, including the undulator emission, can be calculated precisely using classical electrodynamics. Many codes are used in synchrotron light sources, such as *SRW* (Chubar, 1998 ▶), *Spectra* (Tanaka & Kitamura, 2000 ▶) and *XOP* (Sánchez del Río & Dejus, 2011 ▶). For the most common cases they produce similar results. We use *SRW* to compute the undulator power distribution in the plane normal to the beam at the position of the primary slits. Then we make Gaussian fits, and deduce three parameters to describe the power distribution: *P*_a0_, peak power density; and σ_H_ and σ_V_, the horizontal and vertical RMS beam sizes.

Many calorimetry experiments have been carried out to compare the experimental results with the theoretical predictions. The X-ray beam incident power to the LN2-cooled silicon crystal monochromators at the ID06 beamline was first measured in 2004 when the calorimeter described in the previous section was installed, and then in 2010 when the heat load experiments on LN2-cooled silicon crystals were carried out. The power calculated by the *SRW* code takes into account the source parameters: the attenuation by diamond and beryllium windows, the primary slits aperture and the distance from the source. Table 2 ▶ shows the beam power measured by the calorimeter and the comparison with the power calculated using the *SRW* code. The undulators, the aperture of the primary slits and electron beam current were different between the measurements carried out in 2004 and in 2010. The measured beam power is 10–12% smaller than the calculated result. Considering the accuracy of the calorimeter, the power from the undulator in beamline ID06 is about 10% lower than the calculated power. This discrepancy may be explained, for instance, by the accuracy of the primary slits opening and positioning, the accuracy of the thickness of the windows and purity of the window material upstream of the monochromator, the accuracy of the calorimeter, and the accuracy of the undulator parameters (magnetic field, period, gap and length). This discrepancy could be different from one beamline to another, and will be the subject of further investigation. Power losses due to beam scattering on the silicon crystal were estimated to be about 4% of the incident power to the DCM at beamline ID06 according to a Monte Carlo simulation (Secco & Sánchez del Río, 2011 ▶). From the footprint on the first crystal the observing solid angle covered by the second crystal is about 0.4 times half space (calculated from a drawing of the monochromator with the two crystals). The heat load on the second crystal is then about 1.6% of the incident power, and distributed over the surface of the second crystal which is effectively LN2-cooled. The heating and thermal deformation of the second crystal by Compton scattering is therefore negligible. Finally, the power absorbed by the first Si crystal at beamline ID06 is *fp*_cor_ = 14% lower than the calculated power (10 + 4% = 14%). This correction factor will be used to calculate the effectively absorbed power by the first crystal.

In addition, the power absorbed by the first crystal can be estimated as the power evacuated by the LN2 flow in the cooled silicon crystal by measuring the increase of the LN2 temperature Δ*T*_*f*_ between the monochromator outlet and inlet,
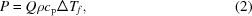
where ρ and *c*_p_ are the density and heat capacity of LN2, and *Q* is the LN2 flow rate in volume per unit of time. In general, this approach provides a direct estimation of the absorbed power. However, it is accompanied by several sources of uncertainty. In particular, the flow rate is estimated from the measured pressure drop. As the pressure drop curve of the LN2 cooling loop *versus* the flow rate is quite flat over a large flow rate range, there can be a rather high degree of uncertainty of the flow rate, *i.e.* in the range 150 to 250 L h^−1^ for the measured pressure drop. This problem can be solved using the results obtained in the laboratory measurements of the temperature increase as a function of the calibrated power load. Another source of uncertainty is the small value of the temperature difference between the outlet flow and inlet flow: 2.7 K for the ID06 monochromator and 3 K for the ID18 monochromator with primary slits opening of 2 mm × 1 mm (H × V) and at 200 mA. Thus, the method based on the measurement of the LN2 temperature difference between the outlet and the inlet of the monochromator is used only for approximate power estimation with an uncertainty of about 20%.

#### 2.3.2. Rocking curve width   

Rocking curves at a Bragg angle of 25.4° were measured using the ID06 beamline monochromator. The Si(333) reflection was selected, because the intrinsic rocking curve width of the double-crystal monochromator at this reflection (*e*_3_ = 13.848 keV) is 6.2 µrad (FWHM), which is significantly smaller than that for the reflection Si(111) (96.2 µrad at 4.616 keV). Thus, the Si(333) reflection at 13.848 keV allows much smaller crystal distortions to be observed.

**Figure 2:**
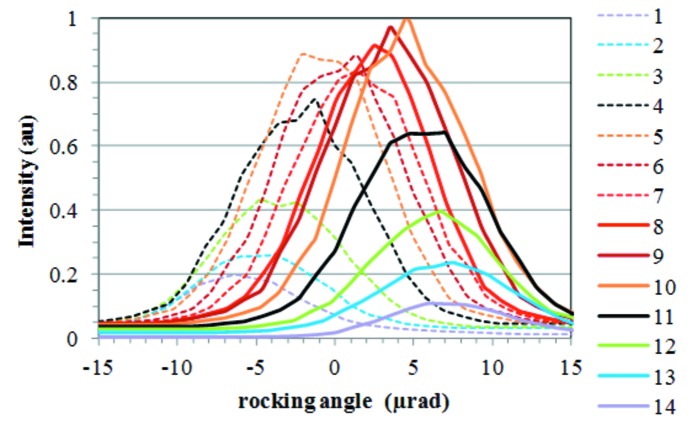
Rocking curves of the monochromator at beamline ID06 for the Si(333) reflection at 13.848 keV, an electron beam current of 101 mA and a power absorbed by the crystal *P* = 194.5 W. The primary slits opening is 2 mm × 1 mm (H × V). The vertical opening of the secondary slit is 0.05 mm. The curves 1 and 14 correspond to the most upstream and downstream position, respectively, on the crystal surface. Each curve (*i*) is measured at a position separated by 0.47 mm from the previous one (*i* − 1) along the beam direction on the crystal surface.

Rocking curves were measured for different positions of a vertically translating narrow-gap slit downstream from the monochromator, as explained in §2.1, and shown in Fig. 2 ▶. The FWHM of these rocking curves is approximately constant (∼9.5 µrad) at an electron beam current *I* = 101 mA and a power absorbed by the crystal *P* = 194.5 W. The thermal slope error including the initial deformation of the crystal is estimated to be 7.2 µrad FWHM (or 3.1 µrad RMS assuming Gaussian distributions) by de-convolving the experimental FWHM with the intrinsic (*i.e.* for an undistorted first crystal) Darwin curve broadening *θ*_intr_ = 6.2 µrad (FWHM). The angular shift of the peak of each rocking curve results directly in the slope of the first crystal at each *x*_c_ coordinate. It is obtained from the peak positions of the Gaussian profiles fitted to the rocking curves. The measured slope profile of the crystal is a weighted average over the transverse footprint width, with the highest weight coefficient along the central axis.

#### 2.3.3. Deformed crystal slope and profiles   

**Figure 3:**
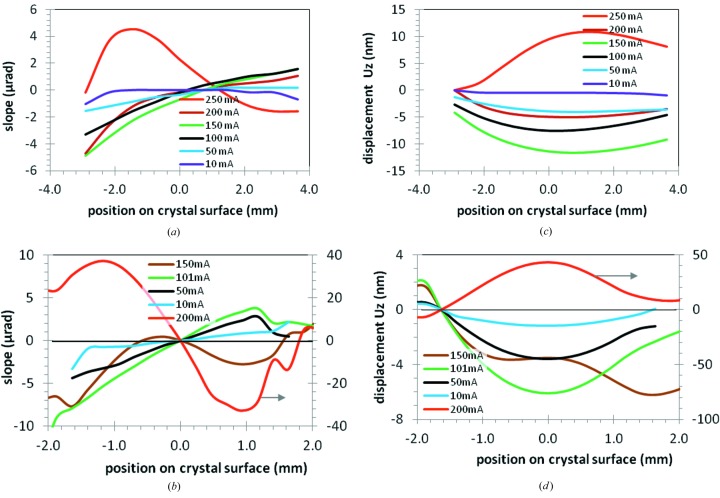
Thermal slope (*a*, *b*) and displacement profile (*c*, *d*) for the first silicon crystal *versus* the crystal coordinate *x*_c_ at various electron beam currents (power load) at the two beamlines ID18 (*a*, *c*) and ID06 (*b*, *d*). The right-hand scale in (*b*, *d*) is for the case of 200 mA electron beam current (red line).

The slope distribution along the crystal footprint length obtained as described above can be used to calculate the deformed crystal profile under the X-ray beam power by the integration of the slope distribution over the crystal coordinate *x*_c_. Results in thermal slope distribution and deformed profile from experiments carried out at beamlines ID18 and ID06 are shown in Figs. 3(*a*)–3(*d*) ▶. At small beam current (low heat load), the shape of the crystal is flat or slightly concave. As the electron beam current (power) is increased, the profile concavity increases, and then decreases to form a mostly flat surface. The electron beam current at which this approximately flat surface is obtained depends on the slit aperture and on the Bragg angle, *i.e.* on both the total power and the power density. When the electron beam current is increased further, the crystal becomes deformed into a convex shape, forming a bump that grows quickly with the electron beam current or heat load on the crystal.

## 3. Finite-element modelling   

The FEA simulations rely on the accurate description of the problem, including the boundary conditions. Several critical issues are discussed here: the material properties of silicon, the beam power absorbed by the crystal, and the finite-element model with mechanical and thermal boundary conditions. The FEA software used is *ANSYS* (release 14.0, ANSYS Inc.).

### 3.1. Material properties   

**Figure 4:**
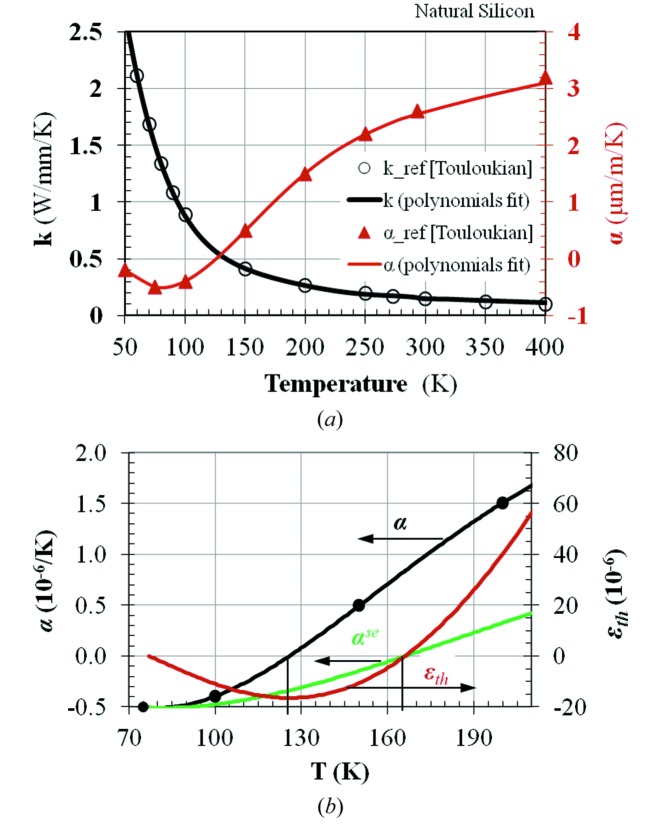
(*a*) Thermal conductivity and thermal expansion coefficient of silicon *versus* temperature. The data represented by circles and triangles are from Touloukian *et al.* (1970*a* ▶); the continuous lines are polynomials fits. (*b*) Thermal expansion coefficients: α (black line), secant α^se^ (green line) and thermal strain ∊_th_ (red line) *versus* temperature for a silicon crystal, the reference temperature being *T*_ref_ = 77 K.

The temperature distribution on the crystal monochromator depends on the impinging X-ray beam power distribution, the thermal conductivity *k* of the crystal material (silicon for the present study), and the crystal geometry and cooling conditions. The temperature gradient induces thermal deformation of the crystal. The related material property is the thermal expansion coefficient α. For materials with constant coefficients *k* and α, the thermal deformation is inversely proportional to the thermal conductivity *k* and proportional to the thermal expansion coefficient α. The ratio α/*k* is often used to estimate the thermal deformation. For silicon, these two material properties are strongly temperature-dependent (Fig. 4*a* ▶). The ratio α/*k* of silicon at LN2 temperature (77 K at 1 atm) is much lower than at room temperature. Therefore, LN2 cooling can significantly reduce the thermal deformation of the silicon crystal compared with water cooling.

The thermal strain of a solid body when the temperature varies from *T*_ref_ to *T* is
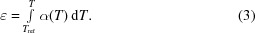
For silicon, the thermal expansion coefficient α is zero at 125 K, but this integral (thermal strain) is not zero when the temperature of the silicon crystal varies from the LN2 temperature (for example, *T*_ref_ = 77 K) to 125 K. When α is constant, this expression becomes
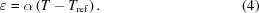
For temperature-dependent α, the strain can still be calculated using a formula similar to (4), but replacing α with the secant coefficient of thermal expansion, α^se^ (see §2.1.3 of the *ANSYS* documentation *Theory Reference*),
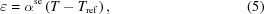
where
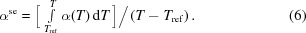
Note that α^se^ depends on both the reference temperature *T*_ref_ and the temperature *T*. The data for the thermal expansion coefficient given in the literature are usually the values of α as a function of temperature, as in Touloukian *et al.* (1970*b* ▶). This is the so-called ‘instantaneous’ α in the *ANSYS* documentation. The secant coefficient of the thermal expansion α^se^ calculated by (6) at *T*_ref_ = 77 K, and the thermal strain ∊_th_ by (3) for silicon are shown as a function of temperature in Fig. 4(*b*) ▶. It is noticeable that the instantaneous coefficient of the thermal expansion α is zero at *T* = 125 K, but the secant coefficient of thermal expansion and the thermal strain are zero at *T* = 165 K. Let us consider a stress-free silicon block cooled down to the LN2 temperature *T*_ref_ = 77 K, then *uniformly* warmed up. From temperature *T*_ref_ = 77 K to 125 K, it is in thermal contraction down to ∊_th_ = −16.5 × 10^−6^ at 125 K [equation (3) or Fig. 4(*b*) ▶ curve ∊_th_]. Above 125 K, the silicon crystal is in thermal expansion that compensates the accumulated thermal contraction from 77 K to 125 K, and then goes back to its initial size (no deformation) at 165 K. For a LN2-cooled silicon crystal under X-ray power, the temperature of the crystal is increased from the initial LN2 temperature to a *higher but not uniform* temperature. The maximum temperature of the crystal is located in the region that is illuminated by the X-ray beam. When this maximum temperature is near *T* = 165 K, the thermal deformation of the crystal reaches a local minimum (but not zero deformation since the temperature in the crystal is not uniform). At *T* = 125 K, the thermal deformation of the silicon crystal is not minimum (see Fig. 4*b* ▶) but mostly thermally contracted. The idea spread throughout the synchrotron radiation community that the LN2-cooled silicon crystal has zero thermal deformation at 125 K is incorrect. (See Appendix *A* [App appa] for a discussion on the *ANSYS* command for the temperature-dependent thermal expansion coefficient.)

The anisotropic mechanical properties of silicon (Wortman & Evans, 1965 ▶; Zhang, 2009 ▶), using the stiffness coefficient matrix for Si(111), have been used in the FEA of this study.

### 3.2. Beam power absorbed by the crystal   

The thermal deformation of the monochromator crystal under heat load depends not only on the material properties as discussed above but also on the beam parameters related to the beam power distribution that are discussed here.

**Figure 5:**
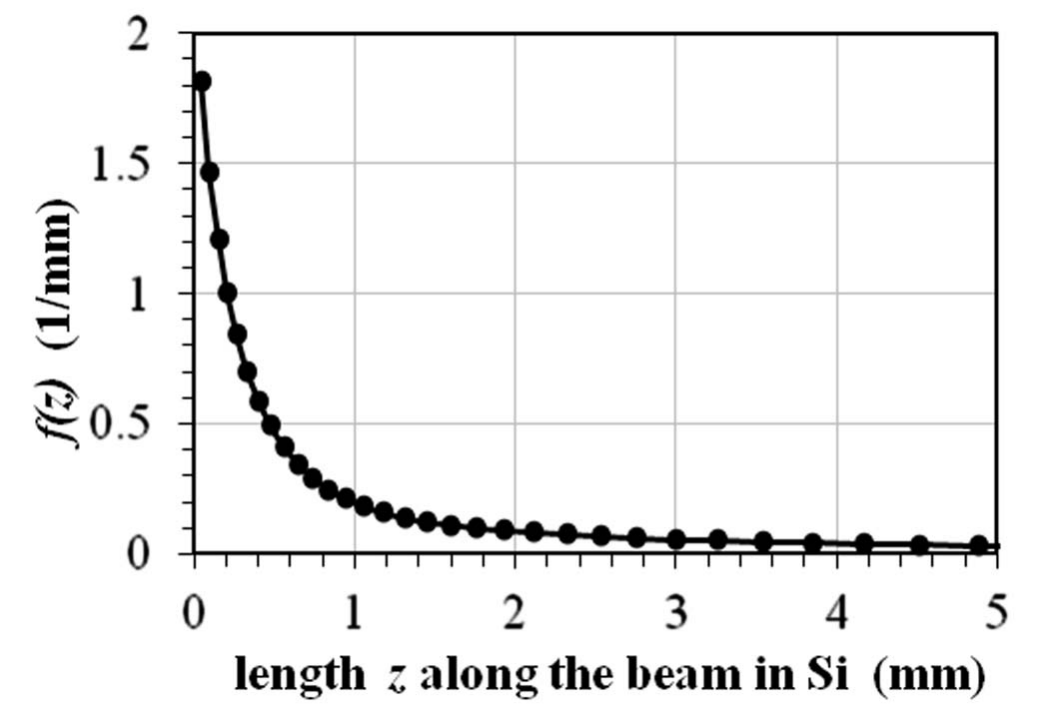
Volume power absorption function *f*(*z*) along the beam path in silicon. The undulator source U18 + U32, a 0.3 mm diamond and a 1 mm-thick beryllium window have been considered in the calculation. This function *f*(*z*) is an attenuation function for silicon averaged over all of the relevant photon energy spectrum up to 100 keV.

For an undulator beam the spatial distribution of the power in a plane normal to the beam at a distance *d* from the source is well approximated by a Gaussian distribution with parameters σ_V_ and σ_H_. In the case of beamline ID06 at the crystal position *d* = 35.8 m from the undulator source, we have σ_V_ = 1.55 mm and σ_H_ = 3.28 mm. Heat load experiments with LN2-cooled silicon crystals have been performed at different electron beam currents. The power *P*_v_ absorbed by a unit volume of the Si crystal at electron beam current *I* is given by
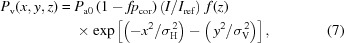
where *P*_a0_ (in W mm^−2^) is the calculated power density at the position of the crystal, *fp*_cor_ = 0.14 is a correction factor for the difference between the calculated power and absorbed power (see §2.3.1), *I*_ref_ is the nominal electron beam current (200 mA), *f*(*z*) is the power absorption function of silicon calculated as the ratio of the absorbed power *P*_abs-by-Δ*z*Si_ by a slice of silicon (thickness Δ*z*) and incident power *P*_inc_ as *f*(*z*) = *P*_abs-by-Δ*z*Si_/*P*_inc_/Δ*z*. The function *f*(*z*) is an attenuation function averaged over the entire photon energy spectrum up to 100 keV. Results of *f*(*z*) are plotted in Fig. 5 ▶. The coordinate system here is *O-xyz*, where *x* and *y* are the horizontal and vertical axes, respectively; the *z*-axis is along the beam path, and the origin of the coordinate system is point *O* at the centre of the footprint on the crystal surface. In a coordinate system lying on the crystal surface *O-XYZ*, a projection factor sinθ_B_ has to be applied accordingly. The units of the different terms are: *P*_v_, W mm^−3^; *P*_a0_, W mm^−2^; *I*, mA; *x*, *y*, σ_H_, σ_V_, mm; *f*(*z*), mm^−1^.

### 3.3. Finite-element model   

**Figure 6:**
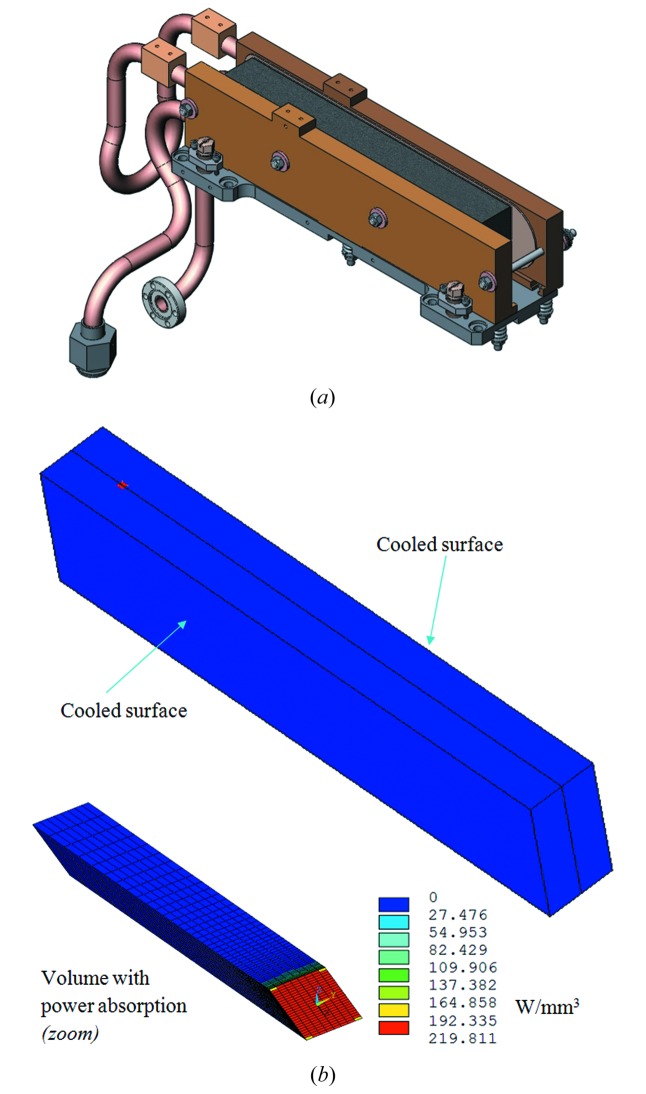
Finite-element model for the ID06 monochromator: (*a*) first silicon crystal with LN2 cooling system, (*b*) finite-element model of the first silicon crystal. The scale for the power density corresponds to a total absorbed power of 385.2 W under the following conditions: primary slits opening = 2 mm × 1 mm (H × V); beam current *I* = 200 mA; power correction factor *fp*_cor_ = 14%.

The first silicon crystal of the ID06 monochromator (as well as those of ID26 and ID18) is cooled from the two sides by copper blocks with a 0.5 mm-thick foil of indium on each of the two interfaces (Fig. 6*a* ▶). The indium foils ensure a good thermal contact between the copper blocks and the silicon crystal. A small pressure maintains them in contact without deforming the crystal. In this study the finite-element modelling is applied only to the silicon crystal, a block 240 mm long, 50 mm thick and 28 mm wide (Fig. 6*b* ▶), as in previous studies (Zhang, 1993 ▶; Zhang *et al.*, 2001 ▶, 2003 ▶). An effective convection cooling coefficient *h*_cv_ is applied to the side surfaces of the crystal in contact with the copper blocks. This cooling coefficient depends on the LN2 flow rate in the cooling blocks and the thermal contact resistance, and is determined from the measured temperature of the crystal. The absorbed power by the volume of the silicon crystal (shown in Fig. 6*b* ▶) is calculated by equation (7) using the function *f*(*z*) shown in Fig. 5 ▶. For the slits opening 2 mm × 1 mm (H × V), an electron beam current of *I* = 200 mA and a power correction factor of *fp*_cor_ = 0.15, the total absorbed power is 385.2 W. At the Bragg angle of θ_B_ = 25.4°, the footprint is close to one end of the crystal as shown in Fig. 6(*b*) ▶, the small red point on the left part of the crystal surface.^1^ The temperature of the crystal is measured at a point on the left end of the crystal close to the bottom, well below the power absorption volume.

The X-ray beam was assumed to impinge along the centre of the crystal, symmetrically on the crystal in the sagittal direction. Therefore, only half of the crystal needs to be modelled. The symmetrical boundary conditions were applied to the central plane parallel to the cooling surfaces. As our interest is in calculating the thermal deformation, it was assumed that the crystal is free of mechanical constraints related to the copper blocks.

The volume of the crystal absorbing the power is an inclined rectangular prism 20 mm in length with the beam footprint (2.58 mm × 3.01 mm) as base (Fig. 6*b* ▶). The footprint is firstly meshed in rectangular two-dimensional elements, and then extruded to three-dimensional elements with progressively increasing element size along the beam path (*z*-axis) over a total length *L*_*z*_ = 20 mm. This corresponds to a silicon thickness (along the *Z*-axis, normal to the crystal surface) of *t*_*Z*_ = 20sinθ_B_ = 8.58 mm. The height of the variable element mesh Δ*Z*_*i*_ is related to the variable thickness Δ*z*_*i*_ used for the calculation of the volume power absorption function as: Δ*Z*_*i*_ = Δ*z*_*i*_sinθ_B_. After a total length of *L*_*z*_ = 20 mm of silicon, the transmitted power (up to 100 keV high-energy photons) is only 3.3% of the incident power. The crystal thickness is 50 mm, which is equivalent to a 116.6 mm length along the X-ray beam path with a Bragg angle of 25.4°. The power transmitted through the whole crystal is negligible (0.01%). In order to limit the size of the model, all the absorbed power is input into the inclined rectangular prism volume. Once this inclined prism volume is meshed, the rest of the silicon crystal is then meshed semi-automatically with several intermediate transition volumes.

## 4. FEA results and comparison with the experiment   

### 4.1. Temperature distribution and cooling coefficient   

The effective cooling coefficient *h*_cv_ depends mostly on the thermal contact resistance at the interface between the silicon crystal and the copper cooling block. The key influencing parameters on this thermal contact resistance are the applied pressure (contact pressure) and the surface state of the contact bodies at the interface (Vallet & Zhang, 2003 ▶; Marion *et al.*, 2004 ▶). Various estimations (Zhang, 1993 ▶; Zhang *et al.*, 2003 ▶) show that this effective cooling coefficient *h*_cv_ for LN2-cooled silicon and copper blocks is mostly in the range 1000–5000 W m^−2^ K^−1^. The exact value of the cooling coefficient *h*_cv_ will be determined when comparing experimental and FEA results.

**Figure 7:**
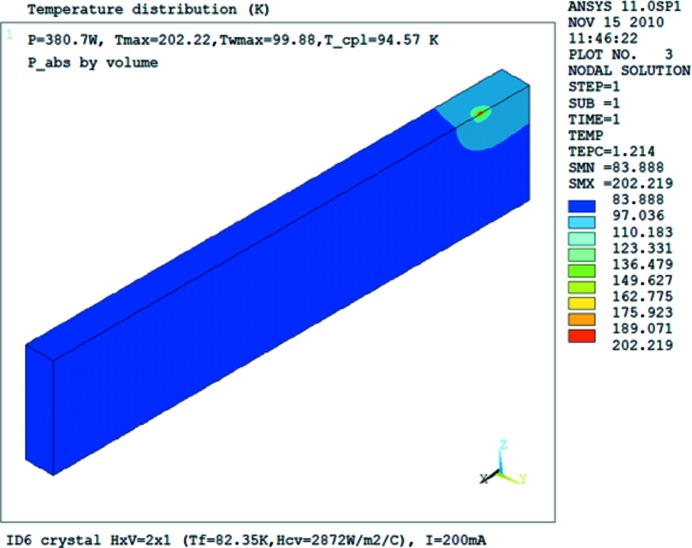
Temperature distribution of the first crystal of the ID06 monochromator for the primary slits opening 2 mm × 1 mm (H × V) and electron beam current *I* = 200 mA (*P* = 385.2 W).

FEA performed by assuming a cooling coefficient *h*_cv_ calculates the temperature distribution in the silicon crystal, in particular at the position where the temperature is measured. The effective cooling coefficient *h*_cv_ can be deduced by fitting the FEA to the measurement temperature at this position. We made this fitting for one heat load case, and then used the deduced cooling coefficient in the calculations for all other heat load conditions. A final fine-tuning of the cooling coefficient *h*_cv_ was carried out to obtain the best temperature fitting. The deduced effective cooling coefficient *h*_cv_ is 2906 W m^−2^ K^−1^ from a first experiment carried out at beamline ID06, and 2656 W m^−2^ K^−1^ from a second experiment performed at the same beamline five months later. The difference between these two values is only 9% and is mostly due to the variation of the LN2 flow rate. The thermal deformation of the LN2-cooled silicon crystal is almost independent of the cooling coefficient when the absorbed power is in the linear region (Zhang *et al.*, 2003 ▶). As an example, the temperature distribution of the silicon crystal is shown in Fig. 7 ▶ for the case of slits opening 2 mm × 1 mm (H × V) and a beam current *I* = 200 mA. The maximum temperature of the crystal in this case reaches 202 K. The region with temperatures higher than 100 K is small and surrounds the beam footprint.

**Figure 8:**
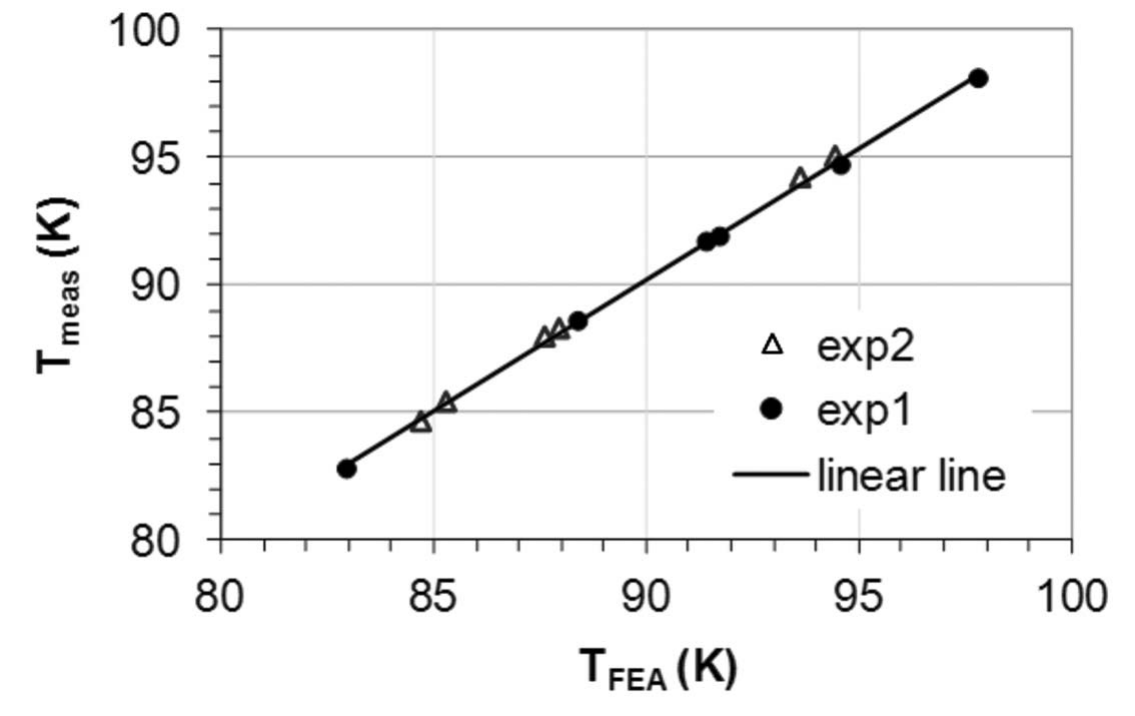
Calculated temperatures by FEA at the position of the thermocouple are compared with the measured ones for different test conditions for the ID06 silicon crystal. Experiment data come from two dedicated heat load test sessions (exp 1 and exp 2). The line is a guide for the eyes. The correlation factor between the calculated and measured temperatures is 0.9995.

The calculated temperatures at the position of the thermocouple are compared with those measured for different test conditions (electron beam current, slits opening) (Fig. 8 ▶). The difference between the calculated and measured temperatures is smaller than 0.6 K. An excellent correlation factor (0.9995) between the calculated temperature and measured temperature confirms the good choice of the thermal boundary conditions (the cooling coefficient and the power load) used in the FEA.

Similar FEA simulations compared with the experimental results at the ID18 beamline led to an effective convective cooling coefficient of *h*_cv_ = 3510 W m^−2^ K^−1^. The mounting of the silicon crystal and copper cooling blocks is not identical in these two beamlines. The contact pressure and the flow rate were also different. Considering these effects, the effective cooling coefficients are comparable between both beamlines ID06 and ID18.

### 4.2. Thermal deformation of the silicon crystal   

The calculated temperature distribution is used to compute the pure thermal deformation in the silicon crystal. It is supposed that the crystal block is free of mechanical constraints although in practice the cooling copper block assembly could introduce mechanical stress and strain. The initial state is chosen to be the crystal shape with mechanical strain due to fabrication/mounting and the cooling down from room temperature to the LN2 temperature just before switching on the heat load. Then, the thermal deformation due to the heat load is superposed linearly to the initial state, and can be separately treated. It is possible to estimate the initial deformation of the crystal by comparing FEA results with the experimental results at a very small power load. As an example, this technique was used to estimate at 1.1 arcsec the initial slope error due to mounting, fabrication and cooling down to LN2 temperature of the channel-cut monochromator crystal at the ESRF beamline ID09 (Zhang *et al.*, 2003 ▶).

**Figure 9:**
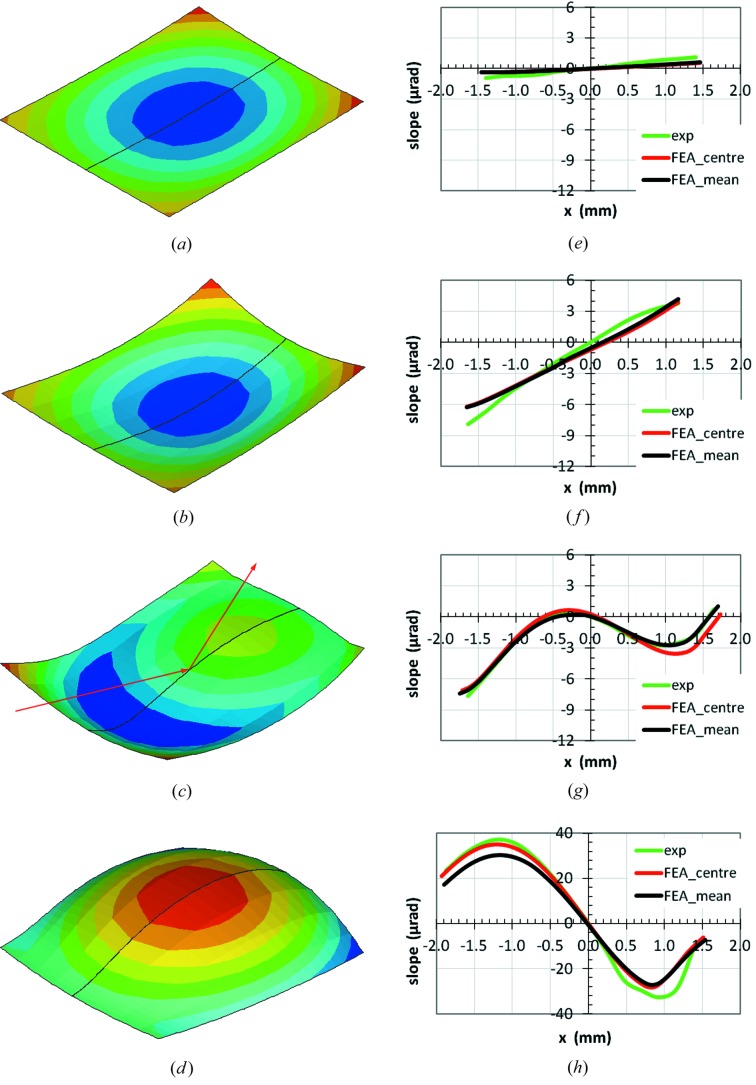
Left column (*a*, *b*, *c*, *d*): FEA results for the deformed crystal shapes in the footprint area with the thermal deformation displacement (the scale of the displacement is different for the four cases) in the direction normal to the crystal surface for four heat load conditions on the crystal at beamline ID06: (*a*, *e*) *P* = 19.1 W, *T*_max_ = 85.0 K, (*b*, *f*) *P* = 194.5 W, *T*_max_ = 120.3 K, (*c*, *g*) *P* = 288.9 W, *T*_max_ = 153.6 K, (*d*, *h*) *P* = 385.2 W, *T*_max_ = 205.7 K. The primary slits opening is 2 mm × 1 mm (H × V). The red lines with arrows show the X-ray beam incident and reflecting directions. Right column (*e*, *f*, *g*, *h*): comparison of calculated and measured thermal slope distributions on the crystal surface in the meridional direction (red line: along the central axis; black line: averaged over the transverse footprint width) for the four heat load conditions. The same scales for the slope distribution are used in (*e*), (*f*) and (*g*); a much larger vertical scale is used for case (*h*).

The pure thermal deformation of the crystal is obtained by assuming that the initial strain in the crystal is negligible and the crystal is flat. The displacement results are directly obtained from FEA for the silicon crystal under power load. The thermal slope along the meridional axis is calculated from the derivative of the displacement *U*_*Z*_ (normal to the crystal surface) to the crystal coordinate *x*_c_ (slope = ∂*U*_*Z*_/∂*x*_c_). For each of these slope profiles within the footprint we calculate the corresponding RMS value. FEA results for the deformed crystal shapes in the footprint area (illuminated by the X-ray beam) are shown in Figs. 9(*a*)–9(*d*) ▶ as well as the thermal deformation displacement *U*_*Z*_ for four representative cases selected from the ID06 beamline measurements. The aperture of the primary slits is 2 mm × 1 mm (H × V). The electron beam current and power load for these four cases are given in Table 3 ▶, which also includes the calculated maximum temperature and RMS slope error along the central axis of the footprint (*x*-axis). On increasing the electron beam current or power load on the crystal, (*a*) at a low absorbed power (19.1 W) the crystal is slightly deformed in a concave shape; (*b*) at a medium absorbed power (194.5 W) the crystal is significantly deformed in a concave shape, the maximum temperature in the crystal is 120.3 K, close to 125 K where the thermal expansion coefficient of silicon is zero, but the thermal strain is negative; (*c*) at a higher absorbed power (288.9 W) the crystal has a shape inversion from concave to convex, the maximum temperature in the crystal is 153.6 K, the RMS thermal slope error is 2.19 µrad, smaller than the 2.98 µrad for case (*b*); (*d*) at an even higher absorbed power (385.2 W) the crystal is very much deformed in a convex shape, the maximum temperature in the crystal is 205.7 K, the RMS thermal slope error is 22.03 µrad. The absorbed power in case (*d*) is only 33% higher than in case (*c*), but the thermal deformation (RMS slope error) is increased by a factor of ten, a very strong non-linear effect. In case (*c*) one observes that the shape inversion from concave to convex occurs on the incident side of the footprint at a smaller absorbed power than on the exit side. This can be explained by the power absorption in the inclined volume with beam incidence at Bragg angle θ_B_ = 25.4°: the volume under the upstream half of the footprint absorbs less power than the volume under the downstream half of the footprint.

The thermal slope distribution from the FEA simulations are also compared with the measurement results for these four cases [Figs. 9(*e*)–9(*h*) ▶]. The FEA results are given for the central axis (FEA_centre), and also averaged over the transverse footprint width (FEA_mean). The averaged slope profile is very close to (though slightly smaller than) the one along the central axis when the silicon crystal is concave (cases *e*, *f*), but significantly smaller when the crystal becomes convex (cases *g* and *h*). As explained in §2.3.2, the measured slope profile of the crystal is a weighted average over the transverse footprint width, with highest weight coefficient along the central axis; the average of the FEA slope distribution should be made with the same weight function. But the latter is unknown. Therefore we use the simple average for FEA results which are in very good agreement with the experimental results in the first three cases (*e*, *f*, *g*). Small discrepancies are found in case (*h*), the case of high heat load and larger thermal deformation. The slope profile along the central axis from FEA is slightly larger than the experimental result in case (*g*), but in good agreement in case (*h*). The accuracy of the measurements of the crystal slope distribution is about 1 µrad. It should be emphasized that the slope distributions of the crystal in the four cases are very different, corresponding to crystal shapes changing from concave to convex, and the FEA simulations agree very well with the measurement results. This good agreement confirms the pertinence of the choice of parameters described in the previous section in FEA. The difference of the thermal slope distribution between experiments and FEA in cases (*e*) and (*f*) shows that the initial shape of the first crystal is concave with a slope error estimated at 1.1 µrad peak-to-peak and about 0.45 µrad RMS.

**Figure 10:**
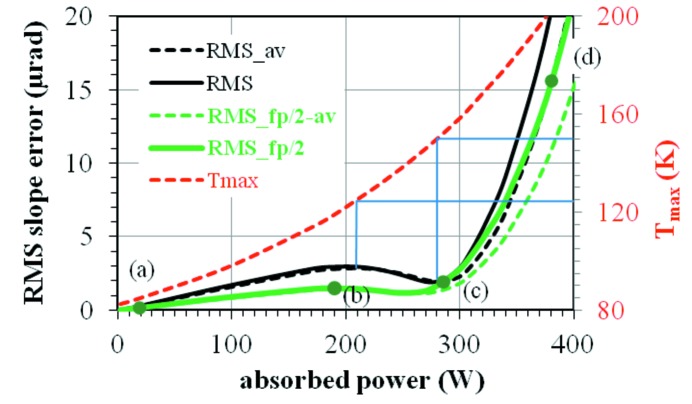
RMS thermal slope error and the maximum temperature of the ID06 LN2-cooled silicon crystal *versus* absorbed power. RMS values are calculated over the whole footprint or half footprint (_fp/2) length, from the slope distribution along the central axis or averaged (_av) over the transverse footprint width. The primary slits opening is 2 mm × 1 mm (H × V). The four points (*a*, *b*, *c*, *d*) correspond to the four heat load conditions shown in Fig. 9 ▶.

For a global picture of the thermally deformed silicon crystal at various heat loads, we have performed FEA simulations as presented above for many values of electron beam current, and plotted the thermal slope error in RMS *versus* absorbed power. For each power load condition, the RMS slope error is calculated from the two slope error profiles (FEA_centre, FEA_mean) described above, which covers the full footprint length defined by the vertical opening of the primary slits. For the ID06 beamline, this vertical opening is 1 mm. The FWHM of the central cone undulator radiation is about 0.5 mm in the vertical at the position of the primary slits, which is half of the primary slit vertical opening. It is also interesting to calculate the RMS slope error over the length corresponding to the projection of the undulator central cone, which is approximately half a footprint length. The RMS thermal slope errors shown in Fig. 10 ▶ are calculated over the whole footprint or half footprint (_fp/2) length, from the slope distribution along the central axis or averaged (_av) over the transverse footprint width. The averaged RMS slope error is very close to the one along the central axis for power values less than 280 W (around the local minimum), but it differs significantly for higher power. The RMS slope calculated over the half footprint length is roughly half of that over the whole footprint length for power smaller than the one corresponding to the local minimum. The four points (*a*, *b*, *c*, *d*) in Fig. 10 ▶ correspond to the four heat load conditions shown in Fig. 9 ▶. The position of these four points in Fig. 10 ▶ and the corresponding crystal shapes shown in Figs. 9(*a*)–9(*d*) ▶ give us a view of different working points within the global behaviour of the RMS slope error and the corresponding deformed shape of the silicon crystal at various heat loads.

The maximum temperature of the crystal *versus* absorbed power is also shown in Fig. 10 ▶. It is about 125 K at the local maximum in the curve of the RMS slope error calculated over the whole footprint. This local maximum corresponds to the most concave shape of the silicon crystal with accumulated thermal contraction effects. Above 125 K the silicon crystal is in thermal expansion. The concave shape, as well as the RMS slope, is reduced to a local minimum in the curve of the slope *versus* the absorbed power. The maximum temperature of the crystal at this local minimum is about 150 K, significantly higher than 125 K but smaller than 165 K (the theoretical *uniform* temperature for non-deformation, as discussed before).

We can also check our FEA results expressed in terms of RMS slope error against experimental results. It is shown in §2.3.2 that the thermal slope error including the initial deformation of the crystal due to mounting is about 7.2 µrad in FWHM (3.1 µrad RMS assuming Gaussian distributions) from the rocking curve width. The initial shape of the first crystal is concave with a slope error estimated to be 0.45 µrad RMS (1.1 µrad peak-to-peak). The pure thermal slope error in RMS should be in the interval (2.65, 3.07) µrad, where the lower and upper limits correspond, respectively, to the cases where the thermal deformed shape and the initial shape of the crystal are perfectly correlated (3.1 − 0.45 = 2.65 µrad) and uncorrelated [(3.1^2^ − 0.45^2^)^1/2^ = 3.07 µrad]. The experimental rocking curves were measured at a power load *P* = 194.5 W [point (*b*) in Fig. 10 ▶]. The pure thermal RMS slope error from FEA is 2.98 µrad along the central axis and 2.86 µrad in a laterally averaged profile, calculated over the whole footprint length (Fig. 10 ▶). Therefore, the estimation of the thermal slope error from the rocking curve broadening measurements is consistent with the FEA results in RMS slope error calculated over the whole footprint length.

**Figure 11:**
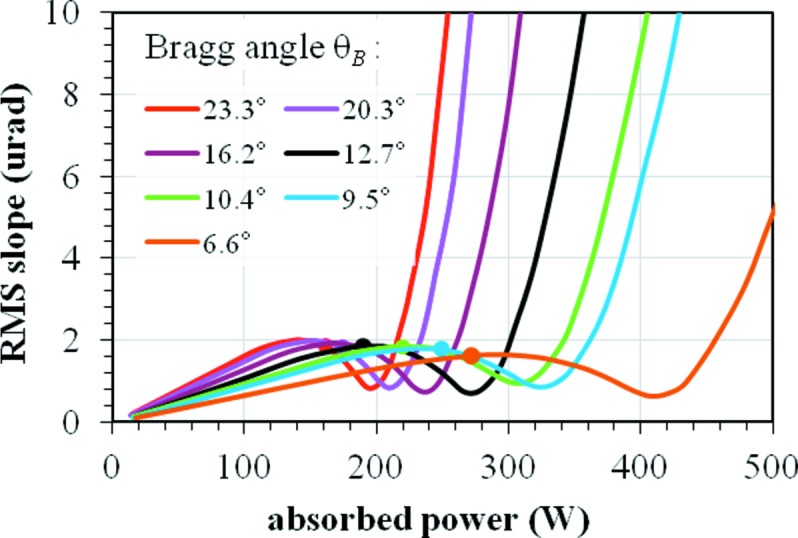
RMS thermal slope error of a LN2-cooled silicon crystal under variable heat load at different Bragg angles for the ESRF UPBL6 beamline project. The RMS thermal slope is calculated over the central cone illuminated length on the crystal surface (half footprint). Working points at *I* = 200 mA (the present most common operation electron beam current at the ESRF) are also shown as circle points.

After being validated by experiments, the FEA simulations can then be used for the design and optimization of beamline optics. An example is the LN2-cooled silicon crystals in the pre-monochromator for the ESRF UPBL6 beamline under construction. The first silicon crystal is a block 150 mm long, 60 mm wide and 100 mm thick, placed at 30 m from the U26 and U32 undulator sources. The photon energy scan range is 5–20 keV, corresponding to Bragg angles between 5.6° and 23.1°. The beam size is defined by the aperture of the primary slits at 27 m: 1.8 mm × 0.8 mm (H × V). The X-ray beam centre cone size is 1.2 mm × 0.4 mm (FWHM_h_ × FWHM_v_). We concentrated on the FEA results in the area illuminated by the centre cone on the crystal surface. The RMS thermal slope error is plotted *versus* absorbed power at various Bragg angles in Fig. 11 ▶. The working points corresponding to an electron beam current of 200 mA (maximum operational current) are indicated in Fig. 11 ▶, and all sit on the flat part (less sensitive to the absorbed power) or left-hand side of the local minimum of the curves, where the silicon crystal has a concave shape. In this zone or at these working points the longitudinal profiles of the crystal are approximately circular and their RMS slope error (proportional to the inverse of the radius of curvature) increases linearly with power. The thermal deformations in this zone can be compensated by other focusing elements in the beamline. Regardless of the angle of incidence, the thermal slope error of the crystal can be kept under 2 µrad. Moreover, it can be lower than 1 µrad if the local minima are chosen as working points with high power load and may be good choices for specific experiments. However, these positions are quite unstable: a small increase in power may result in very high thermal deformations.

**Figure 12:**
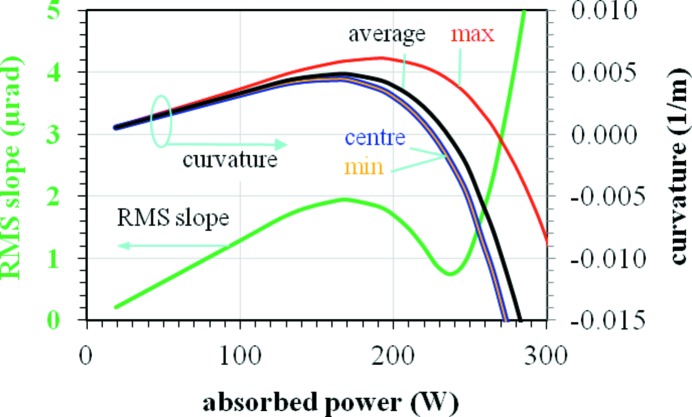
RMS thermal slope (green line, left axis) error *versus* absorbed power at Bragg angle θ_Bragg_ = 16.2° for the LN2-cooled silicon crystal for the ESRF UPBL06 beamline project. Different values of the (non-constant) curvature are plotted (right axis): at the centre of the footprint, the maximum, minimum and average over the central cone illuminated half footprint length. Note that in the absorbed power range (225, 270) W the maximum curvature is positive (concave) whereas the minimum curvature is negative (convex).

The detailed shape of the thermally deformed silicon crystal significantly affects the beam wavefront downstream from the DCM. A plane incident wave can be turned into a converging (diverging) one when the crystal is thermally deformed in a concave (convex) shape. When the crystal shape evolves from concave to convex, the crystal curvature is not constant along the diffracting direction and affects the spatial extension and shape of the wavefront. To estimate the non-uniformity of the curvature along the crystal, the RMS thermal slope error for Bragg angle θ_Bragg_ = 16.2° is plotted *versus* absorbed power (Fig. 12 ▶) together with the curvature at the centre of the footprint, and the maximum, minimum and average curvature over the crystal surface illuminated by the central cone. The average curvature is positive (concave shape) at a small power load, reaches a maximum and then goes through zero at about the same power loads as the local maximum and minimum in the curve of the RMS slope *versus* power, then becomes strongly negative (convex shape or bump) on increasing the absorbed power. The values of the curvature at the centre of the footprint are almost identical to those of the minimum curvature. However, it is worth pointing out that the difference between the maximum and minimum of the curvature indicates that the thermal deformed crystal shape is not spherical (or not constant curvature). Fig. 12 ▶ shows that the curvature induced on the monochromator by the thermal deformation is not uniform and may strongly modify the beam properties, with consequences that clearly depend on the specific beamline design and applications. The plots shown in Figs. 11 ▶ and 12 ▶ are a convenient guide for the design and optimization of the LN2-cooled silicon crystal monochromators, and help to define the beamline optical layout. The results in Fig. 11 ▶ also show that the heat load limit for the LN2-cooled silicon crystal varies strongly with the Bragg angle: 450 W for θ_B_ = 6.6°, 230 W for θ_B_ = 23.1°. These heat load limits depend also on the beam size, but only slightly on the cooling coefficient as reported by Zhang *et al.* (2003 ▶).

## 5. Summary and conclusions   

When measurements of diffracted intensity using a narrow-gap exit slit at various vertical positions are combined with monochromator rocking angle scans, it is possible to measure the thermal deformation profile of LN2-cooled silicon crystals under heat load. This method is very simple, effective and easy to implement with existing beamline components. Up to now, the thermal deformation of the monochromator crystal has been indirectly studied by measuring the rocking curve width broadening, effect of the thermal distortion of the crystal. The method proposed here is a direct measurement of the thermal distortion of the crystal surface. The measurement accuracy depends mostly on the accuracy of the angular positioning and the heat load stability of the rotational stage of the second crystal, and also on the accuracy of the slits, and can be in the range of a fraction of a microradian.

The thermal deformation of LN2-cooled silicon crystal monochromators has been accurately predicted by FEA. The LN2-cooled silicon crystal surface is concave at low heat load, convex at high heat load, and in a complex shape, almost flat on average, at intermediate heat load. The FEA predictions are based on the correct use of material parameters in the FEA software, accurate estimation of the power load on the crystal and relevant boundary conditions including the power absorption and cooling parameters.

The excellent agreement between the measured crystal shapes and FEA simulations under different heat load conditions confirms, once more, the adequacy of using the finite-element modelling for the beamline design and optimization, and validates the use of FEA quantitative results, with a high level of reliability, to fulfil the particular and demanding conditions required by the ESRF Upgrade Programme.

## Figures and Tables

**Figure 13:**
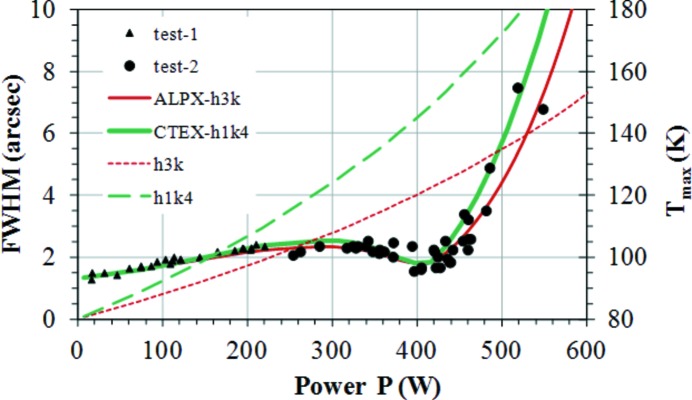
Rocking curve width FWHM and maximum temperature on a channel-cut Si crystal as a function of the total absorbed power. Triangles and circles represent experimental data, the red line ALPX-h3k corresponds to previous calculated rocking curve widths FWHM with a cooling coefficient of 3000 W m^−2^ K^−1^ (Zhang *et al.*, 2003 ▶), the green line CTEX-h1k4 represents recalculated results with the coefficient of thermal expansion input by ‘MP, CTEX,…’. The corresponding maximum crystal temperature curves are the dashed red line h3k, and dashed green line h1k4. Here h1k4 and h3k correspond to cooling coefficients of 1400 and 3000 W m^−2^ K^−1^. The green lines correspond to the corrected FEA results.

**Table 1:** Beamline parameters used in the experiments and some heat load calculation results at 200 mA electron beam current for slits opening of 2 mm × 1 mm (H × V) *d*_src/primary slits_ and *d*_src/mono_ are, respectively, the distance of the primary slits and the monochromator from the undulator source, *P*_total_ and *Pa*_max_ are the total power and the maximum power density of the beam from the undulator source at the position of the primary slits, *P*_total-afterBeD_ and *Pa*_max-afterBeD_ [= *P*_a0_ in equation (7)] are the total power and the maximum power density after windows, filters and just after primary slits. The beam footprint is the projected beam size on the monochromator crystal surface.

Beamline	ID06	ID06	ID06	ID18
Undulator	U18	U32	U18 + U32	3 × U20
Period (mm)	18	32		20
Length (m)	2.0	1.6		4.8
Gap (mm)	8.30	13.55		11
Deflection parameter *K*	0.878	1.636		0.63
Fund energy (keV)	13.848	4.616		14.413
*d*_src/primary slits_ (m)	27.8	27.8	27.8	27
*H*_primary slit_ (mm)	2.0	2.0	2.0	2.0
*V*_primary slit_ (mm)	1.0	1.0	1.0	1.0
Window/filters	0.3 mm diamond + 1 mm Be			0.3 mm diamond
*P*_total_ (W)	352	188	540	438.3
*Pa*_max_ (W mm^−2^)	193.0	98.0	291	256
σ_*x*_ (mm)	1.64	3.66	1.93	1.16
σ_*z*_ (mm)	1.15	1.22	1.17	1.07
*Pa*_max-afterBeD_ (W mm^−2^)	170.9	71.0	241	236
*P*_total-afterBeD_ (W)	311.4	136.3	448	406
*d*_src/mono_ (m)			35.8	30
Bragg angle (°)	25.4	25.4	25.4	7.9
Footprint (H × V) (mm)			2.58 × 3.01	2.22 × 8.08

**Table 2:** Beam power measured by a calorimeter (*P*_meas_) compared with power calculated using the *SRW* code (*P*_SRW_), different electron beam currents (*I*) and primary slits horizontal (H) and vertical (V) apertures The power absorption by windows and filters is considered in the calculated power.

Date	Undulator	H × V (mm)	*I* (mA)	*P*_meas_ (W)	*P*_SRW_ (W)	Difference
2004	U42g12.1	3 × 3	60	163	181	−10%
			85	224	256	−12%

2010	U18g8.3+U32g13.55	2 × 1	100	201	224	−10%
			200	401	448	−10%
			240	475	538	−12%

**Table 3:** Electron beam current (*I*), total power absorbed by the silicon crystal (*P*), calculated maximum temperature (*T*_max_) and RMS slope error along the central axis of the footprint for the four cases shown in Fig. 9 ▶

Case	Electron beam current *I* (mA)	Absorbed power *P* (W)	*T*_max_ (K)	RMS_slope_ (µrad)
(*a*)	9.9	19.1	85.0	0.30
(*b*)	101	194.5	120.3	2.98
(*c*)	150	288.9	153.6	2.19
(*d*)	200	385.2	205.7	22.03

## Footnotes

1 For the ID06 monochromator the centre of rotation is located at half the height between the white and monochromatic beams. Therefore the beam footprint moves across the surface of the first crystal when the Bragg angle is changed. In this geometry it is not necessary to translate the second crystal parallel to the beam. Experiments were carried out at large Bragg angle, where the beam footprint is small and is located relatively close to the centre of rotation, *i.e.* the downstream edge of the crystal.

## Appendix A *ANSYS* command for the temperature-dependent thermal expansion coefficient

In the mathematical formulation used in the *ANSYS* code, the thermal strain is calculated as in equation (4) for constant thermal expansion coefficient α, or using the secant coefficient of the thermal expansion as in equations (4) and (5). The input command is then ‘MP, ALPX,…’. As the thermal expansion coefficient data in the literature are usually reported as a function of temperature (Touloukian *et al.*, 1970*b* ▶), the data should be first converted to the secant form by equation (6), then the command ‘MP, ALPX,…’ can be used for data input.

Since the release of *ANSYS* 8.0, the instantaneous coefficient of the thermal expansion (as given in the literature) can be directly input in *ANSYS* with the command ‘MP, CTEX,…’. The data conversion according to equations (5) and (6) is then made within the *ANSYS* code. In both cases the reference temperature *T*_ref_ is needed. For a LN2-cooled silicon monochromator crystal the reference temperature *T*_ref_ should be the temperature of the cooled crystal before switching on the X-ray power (*T*_ref_ = 77 K for instance). *ANSYS* uses *T*_ref_ = 0 by default, therefore this parameter should always be carefully checked.

There was some confusion on how to input the data of the thermal expansion coefficient when using *ANSYS* code. Before *ANSYS* version 8 (released at the end of 2003), only the secant coefficient of the thermal expansion could be used with the command ‘MP, ALPX,…’. Many *ANSYS* users (including ourselves) have directly input thermal expansion coefficient data from the literature using this command, thus obtaining an incorrect value of the temperature (*T* = 125 K) at which the thermal deformation of the LN2-cooled silicon crystal reaches a local minimum. As a consequence the incorrect idea of thermal deformation being zero at 125 K had been widely spread in the synchrotron community. For silicon the thermal expansion coefficient is zero at 125 K, but the thermal deformation of the LN2-cooled silicon crystal is not zero. The thermal strain of LN2-cooled silicon is zero at 165 K (Fig. 4*b* ▶ in this paper). The X-ray power-induced thermal deformation reaches a local minimum at a maximum temperature of the crystal slightly lower than 165 K. As an example, in the paper by Zhang *et al.* (2003 ▶) *ANSYS* version 6.1 was used and the thermal expansion coefficient from Touloukian *et al.* (1970*b* ▶) was directly input with the only available *ANSYS* command ‘MP, ALPX,…’. We noticed this problem in 2006, and using the correct *ANSYS* command ‘MP, CTEX,…’ for the coefficient of thermal expansion we recalculated the results corresponding to Fig. 8 in the paper (Zhang *et al.*, 2003 ▶), and reported as Fig. 13 ▶ here for the rocking curve widths (FWHM) as well as for the maximum temperature on the Si crystal *versus* total absorbed power. In Fig. 13 ▶ we added two more curves with respect to those appearing in Fig. 8 of that paper (Zhang *et al.*, 2003 ▶): the recalculated rocking curve width FWHM and the maximum temperature of the crystal using the correctly input thermal expansion coefficient by the command ‘MP, CTEX,…’ and with an effective cooling coefficient of 1400 W m^−2^ K^−1^ (green lines). The recalculated rocking curve width FWHM with an effective cooling coefficient of 1400 W m^−2^ K^−1^ fit well with the experimental results reported in that paper. This implies that the effective cooling coefficient was 1400 W m^−2^ K^−1^ instead of 3000 W m^−2^ K^−1^. Therefore, the results published previously are qualitatively correct, and can be further corrected using a smaller effective cooling coefficient. The recalculated results show that the local minimum of the calculated FWHM corresponds to a maximum crystal temperature of about 150 K. This is very close to results reported in Figs. 9 ▶ and 10 ▶ of the present paper.

## References

[bb1] Bilderback, D. H., Freund, A. K., Knapp, G. S. & Mills, D. M. (2000). *J. Synchrotron Rad.* **7**, 53–60. 10.1107/S0909049500000650 16609174

[bb2] Chubar, O. & Elleaume, P. (1998). *Proceedings of the Sixth European Particle Accelerator Conference (EPAC’98)*, pp. 1177–1179.

[bb3] Chumakov, A., Rüffer, R., Leupold, O., Celse, J.-P., Martel, K., Rossat, M. & Lee, W.-K. (2004). *J. Synchrotron Rad.* **11**, 132–141. 10.1107/S0909049503026785 14960777

[bb4] Hoszowska, J., Mocella, V., Zhang, L., Migliore, J. S., Freund, A. & Ferrero, C. (2001). *Nucl. Instrum. Methods Phys. Res. A*, **467**–**468**, 631–634.

[bb5] Lee, W.-K., Fernandez, P. & Mills, D. M. (2000). *J. Synchrotron Rad.* **7**, 12–17. 10.1107/S0909049599014478 16609166

[bb6] Lee, W.-K., Fezzaa, K., Fernandez, P., Tajiri, G. & Mills, D. M. (2001). *J. Synchrotron Rad.* **8**, 22–25. 10.1107/s0909049500013868 11486492

[bb7] Lee, W. K., Mills, D. M., Assoufid, L., Blasdell, R. C., Fernandez, P., Rogers, C. S. & Smither, R. K. (1995). *Opt. Eng.* **34**, 418–425.

[bb8] Marion, P., Zhang, L., Vallet, L. & Lesourd, M. (2004). *Proceedings of MEDSI 2004 – Mechanical Engineering Design of SR Equipment and Instrumentation 2004.* ESRF, Grenoble, France.

[bb9] Marot, G. (1995). *Opt. Eng.* **34**, 426–431.

[bb10] Marot, G., Rossat, M., Freund, A., Joksch, S., Kawata, H., Zhang, L., Ziegler, E., Berman, L., Chapman, D., Hastings, J. B. & Iarocci, M. (1992). *Rev. Sci. Instrum.* **63**, 477.

[bb11] Mocella, V., Ferrero, C., Freund, A., Hoszowska, J., Zhang, L. & Epelboin, Y. (2001). *Nucl. Instrum. Methods Phys. Res. A*, **467**–**468**, 414–417.

[bb12] Mocella, V., Lee, W.-K., Tajiri, G., Mills, D., Ferrero, C. & Epelboin, Y. (2003). *J. Appl. Cryst.* **36**, 129–136.

[bb13] Mochizuki, T., Kohmura, Y., Awaji, A., Suzuki, Y., Baron, A., Tamasaku, K., Yabashi, M., Yamazaki, H. & Ishikawa, T. (2001). *Nucl. Instrum. Methods Phys. Res. A*, **467**–**468**, 647–649.

[bb15] Rogers, C. S., Mills, D. M., Lee, W. K., Knapp, G. S., Holmberg, J., Freund, A., Wulff, M., Rossat, M., Hanfland, M. & Yamaoka, H. (1995). *Rev. Sci. Instrum.* **66**, 2494–2499.

[bb16] Sánchez del Río, M. & Dejus, R. J. (2011). *Proc. SPIE*, **8141**, 814115.

[bb17] Secco, E. & Sánchez del Río, M. (2011). *Proc. SPIE*, **8141**, 81410Z.

[bb18] Tajiri, G., Lee, W.-K., Fernandez, P., Mills, D. M., Assoufid, L. & Amirouche, F. (2001). *J. Synchrotron Rad.* **8**, 1140–1148.

[bb19] Takagi, S. (1962). *Acta Cryst.* **15**, 1311–1312.

[bb20] Takagi, S. (1969). *J. Phys. Soc. Jpn*, **26**, 1239–1253.

[bb22] Tanaka, T. & Kitamura, H. (2000). *SPECTRA – Synchrotron Radiation Calculation Program.* Insertion Device Group, SPring-8, Hyogo, Japan.

[bb23] Taupin, D. (1964). *Bull. Soc. Fr. Minéral. Cristallogr.* **87**, 469–511.

[bb24] Taupin, D. (1967). *Acta Cryst.* **23**, 25–35.

[bb25] Touloukian, Y. S., Kirby, R. K., Taylor, R. E. & Lee, T. Y. R. (1970*a*). *Thermophysical Properties of Matter*, Vol. 13, *Thermal Expansion*, pp. 154–161. New York/Washington: IFI/Plenum.

[bb26] Touloukian, Y. S., Powell, R. W., Ho, C. Y. & Klemens, P. G. (1970*b*). *Thermophysical Properties*, Vol. 1, *Thermal Conductivity – Metallic Elements and Alloys*, pp. 326–339. New York/Washington: IFI/Plenum.

[bb27] Vallet, L. & Zhang, L. (2003). *Résistance thermique de contact.* Internal report. ESRF, Grenoble, France.

[bb28] Wortman, J. J. & Evans, R. A. (1965). *J. Appl. Phys.* **36**, 153.

[bb29] Zhang, L. (1993). *Proc. SPIE*, **1997**, 223–235.

[bb30] Zhang, L. (2009). *AIP Conf. Proc.* **1234**, 797–800.

[bb31] Zhang, L. & Biasci, J. C. (2005). *Heatload tests on ID6*, ESRF Annual Machine Workshop. ESRF, Grenoble, France.

[bb32] Zhang, L., Hoszowska, J., Migliore, J. S., Mocella, V., Ferrero, C. & Freund, A. (2001). *Nucl. Instrum. Methods Phys. Res. A*, **467**–**468**, 409–413.

[bb33] Zhang, L., Lee, W.-K., Wulff, M. & Eybert, L. (2003). *J. Synchrotron Rad.* **10**, 313–319. 10.1107/s0909049503012135 12824931

